# Carotid Intima-Media Thickness and Visit-to-Visit HbA1c Variability Predict Progression of Chronic Kidney Disease in Type 2 Diabetic Patients with Preserved Kidney Function

**DOI:** 10.1155/2016/3295747

**Published:** 2016-12-20

**Authors:** Akiko Takenouchi, Ayaka Tsuboi, Miki Kurata, Keisuke Fukuo, Tsutomu Kazumi

**Affiliations:** ^1^Department of Food Sciences and Nutrition, School of Human Environmental Sciences, Hyogo, Japan; ^2^Research Institute for Nutrition Sciences, Mukogawa Women's University, Hyogo, Japan; ^3^Department of Nutrition, Osaka City Juso Hospital, Osaka, Japan; ^4^Diabetes Division, Sadamitsu Hospital, Hyogo, Japan

## Abstract

*Background/Aims*. Subclinical atherosclerosis and long-term glycemic variability have been reported to predict incident chronic kidney disease (CKD) in the general population. However, these associations have not been investigated in patients with type 2 diabetes with preserved kidney function.* Methods*. We prospectively followed up 162 patients with type 2 diabetes (mean age, 62.3 years; 53.6% men) and assessed whether carotid intima-media thickness (IMT) measured by B-mode ultrasound and visit-to-visit HbA1c variability are associated with deterioration of CKD (incident CKD defined as estimated GFR [eGFR] < 60 mL/min/1.73 m^2^ and progression of CKD stages) over a median follow-up of 6.0 years. At baseline, 25 patients (15.4%) had CKD. Cox proportional hazards regression models were used for identifying associated factors of CKD deterioration.* Results.* Estimated GFR decreased from 75.8 ± 16.3 to 67.4 ± 18.2 mL/min/1.73 m^2^ (*p* < 0.01). Of 162 patients, 32 developed CKD and 8 made a progression of CKD stages. Multivariate Cox regression analysis revealed that carotid IMT (HR: 4.0, 95% CI: 1.1–14.226.7, and *p* = 0.03) and coefficient of variation of HbA1c (HR: 1.12, 95%: 1.04–1.21, and *p* = 0.003) were predictors of deterioration of CKD independently of age, mean HbA1c, urinary albumin/creatinine ratio, baseline eGFR, uric acid, and leucocyte count.* Conclusions.* Subclinical atherosclerosis and long-term glycemic variability predict deterioration of chronic kidney disease (as defined by incident or worsening CKD) in type 2 diabetic patients with preserved kidney function.

## 1. Introduction

Chronic kidney disease (CKD) has been consistently and independently associated with longitudinal risk for cardiovascular disease and heart failure. Progression towards end-stage renal disease exposes CKD patients to an increased risk of development of vascular disease and cardiovascular morbidity and mortality [1, 2]. Established cardiovascular disease risk factors (diabetes and hypertension) are associated with the development of new-onset kidney disease [3]. Other cardiovascular disease risk factors, such as cigarette smoking, inflammation, and dyslipidemia, have also been linked to declining kidney function in some, but not all, studies [4–8].

These overlapping risk factor patterns lead us to hypothesize that atherosclerosis may be an important mechanism leading to declines in kidney function. Prospective studies have shown a positive correlation between increased carotid artery intima-media thickness (IMT), a subclinical marker of atherosclerosis and cardiovascular disease, and the risk for kidney function decline and incident CKD in the elderly [9, 10]. In addition, carotid IMT was associated with incident CKD in the general population [11]. However, there is limited understanding of whether the presence of a subclinical marker of atherosclerosis, like carotid IMT, is an important predictor for progression to CKD in type 2 diabetic patients. We hypothesized that, among patients with type 2 diabetes and normal or near-normal kidney function at the initiation of the observation period, the baseline IMT would be associated with the deterioration of CKD over time. We have recently shown a direct association between visit-to-visit HbA1c variability and kidney function decline in patients with type 2 diabetes [12]. Hence, we examined in the present study an association between visit-to-visit HbA1c variability and deterioration of CKD as well.

## 2. Patients and Methods

The setting for this observational study was the same as previously reported [12]. We report here results of 162 patients in whom carotid IMT was measured during the first 12 months after enrollment. They had been regularly attending the clinic in 2004 and 2005. They were enrolled in the study at the first visit in 2005 and followed up for the subsequent at least 24 months through December 31, 2012, to assess kidney function with a median follow-up of 6.0 years (interquartile range: 4.1–6.5 years). There were 14 patients who had been regularly attending the clinic in 2004 and 2005 and did not follow up for the subsequent 24 months. One patient died of a traffic accident, 6 moved, and 3 patients stopped visiting because of their job situations. The reason was not known in the remaining 4 patients. There were no differences between the 14 patients and 162 patients studied in anthropometric, clinical, and biochemical variables (data not shown). Patients with hepatitis B surface antigen or antibodies against hepatitis C virus were excluded. Those who had aspartate aminotransferase and alanine aminotransferase of 100 U/L or greater and serum creatinine ≥ 2.0 mg/dL were excluded as well. Study protocol was consistent with the Japanese Government's Ethical Guidelines Regarding Epidemiological Studies in accordance with the Declaration of Helsinki.

For each subject on each monthly visit, waist circumference, weight, and BP were measured by registered nurses. As previously reported in detail [12], blood was withdrawn on 2 occasions: at 2 h after breakfast taken at home and after an overnight fasting in the majority of patients (94%). This was done every other month. Plasma glucose (PG), serum cholesterol, triglyceride (TG), HDL cholesterol, creatinine, and uric acid were measured by standard methods using an autoanalyzer. HbA1C values were determined by high performance liquid chromatography. LDL cholesterol was calculated by Friedewald's formula using lipid levels obtained in blood samples taken after an overnight fasting. Complete blood cell count was analyzed using an automated blood cell counter.

Intrapersonal mean and coefficient of variation (CV) of HbA1c, fasting and postmeal plasma glucose (FPG and PMPG, resp.), and serum TG (FTG and PMTG, resp.) taken during the first 12 months after enrollment were calculated and their means ± SD were shown in Table 1; 153 patients (94%) had 6 measurements of LDL cholesterol, FPG, PMPG, FTG, and PMTG and 12 measurements of HbA1c and systolic BP.

Urinary albumin was measured once in random urine samples obtained during the first 3-4 months after enrollment using a turbidimetric immunoassay and expressed as albumin/creatinine ratio (ACR). Normoalbuminuria, microalbuminuria, and macroalbuminuria were defined as ACR < 30 mg/g, ACR between 30 and 299 mg/g, and ACR ≥ 300 mg/g, respectively [13]. Serum and urinary creatinine were measured enzymatically and estimated glomerular filtration rate (eGFR) was determined using the equation recommended by the Japanese Society for Nephrology [14]. CKD was diagnosed on 2 occasions: one at the entry and the other at the end of the observation time period. CKD was diagnosed using eGFR based on 2–4 measurements of serum creatinine obtained during 3-4 months on the 2 occasions. After the first visit in 2005 they were followed up for the subsequent 24 months and longer through December 31, 2012, with a median follow-up of 6.0 years (interquartile range: 4.1–6.5 years). Incident CKD was defined as eGFR < 60 mL/min/1.73 m^2^ after 6.0 years in participants with an eGFR > 60 mL/min/1.73 m^2^ at baseline. Linear regression was used to estimate changes in eGFR using a median of 52 creatinine measurements (interquartile range: 31–60) over 6.0 years of follow-up in each patient. Patients were staged according to the level of eGFR in mL/min/1.73 m^2^: G1: >90, G2: 60–89, G3a: 45–59, G3b: 30–44, and G4: 15–29 [15].

IMT was measured by a well-trained medical technologist of Sadamitsu Hospital using ultrasonic diagnosis equipment (Shimadzu SDU-2200, Shimadzu, Tokyo, Japan) that was programmed with IMT software (Intimascope, Media Cross Co. Ltd., Tokyo, Japan) as previously described [16]. Carotid artery ultrasonography was performed using a 10-MHz scanning frequency in B-mode with the participant in the supine position. Computer-based IMT was evaluated by two methods: maximum and average evaluations. Maximum evaluation was obtained by the IMT value at a maximal point of the region. Mean IMT is the average value of 250 computer-based points in the region. Mean values of the right and left maximum IMT and mean IMT were used for statistical analysis. The intraobserver CV for IMT measurements was 5.6 ± 0.8% and interobserver CV ranged from 2.5 to 10.9% with an average of 5.9% [16].

Data were presented as means ± SD unless otherwise stated. Differences between 2 groups were analyzed by *t*-test and frequencies of conditions by chi-square tests. Bivariate and multivariable Cox proportional hazards regression models were used for identifying associated factors of deterioration of CKD. Results were expressed as hazard ratio (HR) with their 95% confidence intervals (CI). A two-tailed *p* < 0.05 was considered statistically significant. All calculations were performed with SPSS system 15.0 (SPSS Inc., Chicago, IL).

## 3. Results

The baseline characteristics of 162 participants are shown in Table 1. Patients had relatively good glycemic, lipid, and BP control as previously reported [12]. Glucose-lowering therapy such as GLP-1 agonists, DPP4 inhibitors, and SGLT2 inhibitors is reported to improve renal function in type 2 diabetes patients. Among these drugs, however, a DPP4 inhibitor became available at the beginning of 2010 in Japan. There were only 6 patients who had been on the DPP4 inhibitor for the last 18 months of the study. Means of age and duration of diabetes in Tables 1 and 2 were those on study enrollment. Means of serum creatinine and eGFR were means of 2–4 measurements during the first 3-4 months after enrollment. ACR was measured once during the first 3-4 months after enrollment. Means of other variables were means of the intrapersonal mean values during the first 12 months after enrollment.

As compared to patients without CKD, those with CKD at baseline were older and had longer duration of diabetes and higher proportion of patients on BP-lowering agents (Table 1). Urinary ACR was higher in CKD patients. Although overt proteinuria was more prevalent in patients with than in those without CKD (15 versus 1%, *p* = 0.001) there was no difference in the proportion of patients with microalbuminuria (30 and 28% in patients with and without CKD, resp.). Consequently, patients with CKD tended to have higher proportion of ACR ≥ 30 mg/g (Table 1). Diastolic BP was lower in CKD patients although the 2 groups did not differ in mean and CV of systolic BP. Fasting and postmeal TG were higher and HDL and LDL cholesterol were lower in patients compared to those without CKD although all differences were of borderline significance (*p* = 0.06). There was no difference in glycemic variables and carotid IMT between the 2 groups.

The level of eGFR decreased from 75.8 ± 16.3 mL/min/1.73 m^2^ at baseline to 67.4 ± 18.2 mL/min/1.73 m^2^ after 6.0 years of follow-up (*p* < 0.01) with an annual decline of 1.11 ± 2.94 mL/min/1.73 m^2^. Proportion of patients with CKD (eGFR < 60 mL/min/1.73 m^2^) increased from 15.5% (*n* = 26) at baseline to 32.7% (*n* = 55) after follow-up whereas proportion of patients with stage G2 decreased from 63.6% (*n* = 107) to 54.7% (*n* = 92) and proportion with G1 decreased from 20.9% (*n* = 35) to 12.5% (*n* = 21).

Incident CKD (eGFR < 60 mL/min/1.73 m^2^) occurred in 32 patients. Progression from stage 3A to stage 3B and stage 4 occurred in 7 patients and 1 patient, respectively. In total deterioration of CKD occurred in 40 patients (Table 2). There were 3 patients whose CKD stage improved from G3A to G2 (eGFR: 60–75). They were included as nonprogressors. Patients compared with those without CKD deterioration were older and had higher uric acid levels. Baseline eGFR was lower while annual eGFR decline was higher in patients with CKD deterioration. Carotid maximum and mean IMT, urinary ACR, and leucocyte count also were higher in patients with CKD deterioration whereas the 2 groups did not differ in the proportion of patients with ACR ≥ 30 mg/g. Although, among 162 patients, those on RAS inhibitors increased in number from 68 at baseline to 92 at the end of the study, the percentage of those patients increased at a similar rate both in progressors and in nonprogressors (from 40% to 60% and 43% to 56%, resp.).

Cox proportional hazard regression models were used for identifying associated factors of deterioration of CKD (Table 3). Independent variables included were 6 variables that showed significant associations with CKD progression in Table 1. Mean and CV of HbA1c were also included because they showed significant associations with CKD progression in bivariate model (HR: 1.5, 95% CI: 1.1–2.0, and *p* = 0.006 and HR: 1.09, 95% CI: 1.09–1.13, and *p* < 0.001, resp.). Maximum IMT and CV HbA1c were significant predictors of CKD deterioration. Leucocyte count and baseline eGFR were associated with CKD deterioration, but the relations were of borderline significance. In a model which included mean IMT instead of maximum IMT, CV-HbA1c (HR: 1.12, 95% CI: 1.04–1.21, and *p* = 0.004), mean IMT (HR: 7.8, 95% CI: 0.8–76, and *p* = 0.08), baseline eGFR (HR: 0.97, 95% CI: 0.94–1.002, and *p* = 0.07), and leucocyte count (HR: 1.29, 95% CI: 0.97–1.71, and *p* = 0.08) were independent predictors of CKD deterioration.

## 4. Discussion

This study demonstrates in type 2 diabetic patients with preserved kidney function and relatively good glycemic, lipid, and BP control at baseline that the presence of CVD measured as subclinical carotid atherosclerosis was a predictor for deterioration of CKD. In addition, a nontraditional risk factor, namely, visit-to-visit variation in HbA1c, was found to be a risk factor of CKD deterioration.

Cross-sectional associations between reduced kidney function and IMT thickening are reported in patients with type 2 diabetes [17, 18], hypertension [19], and CKD [20, 21] and in outpatients [22]. Prospective studies have shown a positive correlation between increased carotid IMT and the risk for kidney function decline and incident CKD in the elderly and the general population [9–11]. Recently, carotid IMT has been reported to be associated with end-stage renal disease in the general population [23]. The current study is the first, to our knowledge, to report that subclinical carotid atherosclerosis is an independent predictor of future deterioration of CKD in type 2 diabetic patients with preserved kidney function. These findings suggest the shared etiology of atherosclerosis and progression of kidney function.

Long-term glycemic variability has been suspected to be a major factor of diabetic complications [24]. We have recently shown a direct association between visit-to-visit HbA1c variability and an annual decline in eGFR of patients with type 2 diabetes [12]. In the present study, the association between visit-to-visit HbA1c variability and the progression of CKD was confirmed. Several reasons may be involved in the association between visit-to-visit HbA1c variability and outcomes as suggested by Ceriello and Kilpatrick [25]. They include “metabolic memory” phenomenon [26]. They may be related to the fact that microvascular complication risk rises exponentially, rather than linearly, as HbA1c rises. It is also possible that patient with HbA1c variability are those in whom the rest of their diabetes management is suboptimal.

There were no differences in BP between patients whose kidney function deteriorated and those whose kidney function did not in the present study. This may be in line with observation that BP control of 138/86 or 132/78 mmHg appeared to stabilize renal function in hypertensive type 2 diabetic patients without overt albuminuria over a 5-year period [27], as progressors and nonprogressors in the present study had BP control of 131/72 and 128/72 mmHg, respectively.

The strength of the current study is that mean and CV of HbA1c and other traditional and nontraditional risk factors shown in Table 1 were calculated from 6–12 measurements in 94% of participants. In addition, the diagnosis of CKD was based on 2–4 creatinine measurements not only at entry but also at the end of the observation. Therefore, participants who met the criteria did so for a period of at least 3 months. As previously described in detail [12], serum creatinine and hence eGFR during follow-up period were much more frequently measured. This could contribute to the reliability of changes in kidney function. Major limitations are that study participants were small in number and from a single clinic in Japan. However, the characteristics of our study participants are similar to those reported in a previous large-scale study in Japan [28]. We used eGFR rather than more precise measures of kidney function, like iothalamate clearance. In addition, this cohort of participants consisted of Japanese only, which limits generalizability. Finally, the sample size and the number of CKD related incidents occurred in the present study seem to be small to lead a robust conclusion although statistical power was not calculated.

## 5. Conclusions

Measurements of carotid IMT and visit-to-visit HbA1c variability retain predictive power with respect to CKD progression even after traditional and nontraditional risk factors for kidney disease progression have been taken into consideration. These results suggest that type 2 diabetic patients with subclinical carotid atherosclerosis should be recognized as a group at high risk for CKD progression. In addition, these findings also raise the issue of whether aggressive treatment of traditional risk factors for subclinical carotid atherosclerosis might, in fact, decrease progression to renal replacement therapy. Further studies are needed to confirm the association in other ethnic groups with more patients.

## Figures and Tables

**Table 1:** Baseline characteristics of 162 patients with type 2 diabetes who had and did not have chronic kidney disease at baseline.

	Overall	Chronic kidney disease at baseline		*p* values
	(*n* = 162)	Present (*n* = 25)	Absent (*n* = 137)	
Male sex (*n*, %)	89, 55	12, 48	77, 56	0.45
Smokers (*n*, %)	53, 33	6, 24	47, 3	0.30
Age (years)	62 ± 10	69 ± 9	61 ± 10	0.000
BMI (kg/m^2^)	24.2 ± 3.7	25.3 ± 4.0	24.0 ± 3.6	0.10
Waist circumference (cm)	87.0 ± 10.0	88.8 ± 7.7	86.7 ± 10.3	0.35
Duration of diabetes (years)	9.7 ± 7.2	12.8 ± 8.0	9.1 ± 7.0	0.02
Treatment of diabetes; diet/OHA/insulin (*n*, %)	53/81/28, 33/50/17	4/14/7, 16/56/28	49/67/21, 36/49/15	0.10
Hypertension; CCB/RASi/diuretics (*n*, %)	56/68/7, 35/42/4	18/17/5, 72/68/20	38/51/2, 28/37/1	0.000
HbA1c (%)	7.0 ± 0.8	7.2 ± 0.8	7.0 ± 0.9	0.25
Fasting PG (mg/dL)	125 ± 23	125 ± 24	126 ± 22	0.86
Postbreakfast PG (mg/dL)	154 ± 48	162 ± 51	152 ± 48	0.37
CV-HbA1c (%)	6.7 ± 5.7	6.8 ± 4.5	6.7 ± 5.9	0.96
Total cholesterol (mg/dL)	188 ± 21	178 ± 26	190 ± 20	0.012
LDL cholesterol (mg/dL)	112 ± 22	104 ± 24	113 ± 22	0.06
HDL cholesterol (mg/dL)	55 ± 15	50 ± 12	56 ± 16	0.06
Fasting TG (mg/dL)	114 ± 51	132 ± 73	111 ± 45	0.06
Postbreakfast TG (mg/dL)	145 ± 62	167 ± 80	141 ± 57	0.06
Serum creatinine (mg/dL)	0.8 ± 0.2	1.0 ± 0.3	0.7 ± 0.1	0.000
eGFR (mL/min/1.73 m^2^)	76 ± 16	51 ± 9	80 ± 13	0.000
ΔeGFR (mL/min/1.73 m^2^/year)	−1.1 ± 2.9	−1.2 ± 2.4	−1.1 ± 3.0	0.93
Uric acid (mg/dL)	5.2 ± 1.3	5.7 ± 1.3	5.1 ± 1.2	0.03
Systolic BP (mmHg)	128 ± 12	131 ± 12	128 ± 12	0.20
CV-systolic BP (%)	8.1 ± 2.2	8.4 ± 2.1	8.0 ± 2.2	0.41
Diastolic BP (mmHg)	72 ± 6	69 ± 5	73 ± 7	0.02
Urinary ACR (mg/g)	86 ± 327	233 ± 531	59 ± 268	0.014
log ACR	1.3 ± 0.6	1.7 ± 0.8	1.2 ± 0.5	0.001
Leucocyte count (10^3^/*μ*L)	5.8 ± 1.5	6.1 ± 1.3	5.7 ± 1.5	0.19
Maximum IMT (mm)	1.0 ± 0.3	1.1 ± 0.4	1.0 ± 0.3	0.14
Mean IMT (mm)	0.8 ± 0.2	0.9 ± 0.2	0.8 ± 0.2	0.26
ACR ≥ 30 mg/g (*n*, %)	53, 33	12, 48	41, 30	0.08

Mean ± SD or *n*, %. OHA; oral hypoglycemic agents, CCB; calcium channel blockers, RASi; renin-angiotensin system inhibitors, PG; plasma glucose, CV; coefficient of variation, eGFR; estimated glomerular filtration rate, ΔeGFR; annual changes in eGFR, BP; blood pressure, ACR; albumin/creatinine ratio, IMT; intima-media thickness, ^*∗*^*p* < 0.05, ^*∗∗*^*p* < 0.01, and ^*∗∗∗*^*p* < 0.001.

**Table 2:** Baseline clinical and biochemical characteristics of 162 type 2 diabetic patients whose kidney function deteriorated and did not deteriorate over a 6.0-year follow-up.

	Deterioration		*p* values
	Present (*n* = 40)	Absent (*n* = 122)	
Male sex (*n*, %)	21, 53	68, 56	0.72
Smokers (*n*, %)	14, 35	39, 32	0.75
Age (years)	66 ± 10	61 ± 10	0.01
BMI (kg/m^2^)	24.6 ± 3.1	24.1 ± 3.9	0.52
Waist circumference (cm)	87.9 ± 7.8	86.7 ± 10.6	0.52
Duration of diabetes (years)	10.6 ± 8.1	9.4 ± 7.0	0.35
Treatment of diabetes; diet/OHA/insulin (*n*, %)	12/21/7, 30/53/18	41/60/21, 34/49/17	0.91
Hypertension; CCB/RASi/diuretics (*n*, %)	17/16/3, 43/40/8	39/52/4, 32/43/3	0.41
HbA1c (%)	7.1 ± 0.8	7.0 ± 0.9	0.42
Fasting PG (mg/dL)	125 ± 22	125 ± 23	0.97
Postbreakfast PG (mg/dL)	155 ± 44	154 ± 50	0.87
CV-HbA1c (%)	7.9 ± 5.7	6.4 ± 5.7	0.15
Total cholesterol (mg/dL)	188 ± 21	188 ± 21	0.998
LDL cholesterol (mg/dL)	112 ± 22	111 ± 22	0.92
HDL cholesterol (mg/dL)	53 ± 14	56 ± 16	0.28
Fasting TG (mg/dL)	127 ± 55	110 ± 49	0.06
Postbreakfast TG (mg/dL)	158 ± 70	141 ± 59	0.17
Serum creatinine (mg/dL)	0.8 ± 0.2	0.7 ± 0.2	0.07
eGFR (mL/min/1.73 m^2^)	69 ± 14	78 ± 17	0.004
ΔeGFR (mL/min/1.73 m^2^/year)	−3.1 ± 2.9	−0.5 ± 2.7	0.000
Uric acid (mg/dL)	5.6 ± 1.5	5.1 ± 1.2	0.048
Systolic BP (mmHg)	131 ± 10	128 ± 12	0.17
CV-systolic BP (%)	8.1 ± 2.3	8.0 ± 2.2	0.82
Diastolic BP (mmHg)	72 ± 6	72 ± 7	0.85
Urinary ACR (mg/g)	223 ± 624	41 ± 89	0.002
log ACR	1.5 ± 0.6	1.2 ± 0.5	0.013
Leucocyte count (10^3^/*μ*L)	6.2 ± 1.4	5.7 ± 1.5	0.046
Maximum IMT (mm)	1.1 ± 0.3	1.0 ± 0.3	0.03
Mean IMT (mm)	0.9 ± 0.2	0.8 ± 0.2	0.02
ACR ≥ 30 mg/g (*n*, %)	17, 43	36, 30	0.13

Mean ± SD or *n*, %. Abbreviations are the same as in Table 1.

**Table 3:** Cox regression analyses for exacerbation of chronic kidney disease in patients with type 2 diabetes and preserved kidney function.

Independent variables	HR	95% CI	*p* values
Age	1.032	0.976–1.091	0.27
HbA1c	1.256	0.732–2.154	0.41
CV-HbA1c	1.121	1.040–1.208	0.003
log ACR	1.800	0.838–3.868	0.13
Maximum IMT	4.020	1.135–14.24	0.03
eGFR	0.971	0.942–1.001	0.06
Uric acid	1.124	0.798–1.582	0.50
Leucocyte count	1.304	0.979–1.737	0.07

HR: hazard ratio. CI: confidence interval. Other abbreviations are the same as in Table 1.

## References

[1] Manjunath G., Tighiouart H., Ibrahim H. (2003). Level of kidney function as a risk factor for atherosclerotic cardiovascular outcomes in the community. *Journal of the American College of Cardiology*.

[2] Keith D. S., Nichols G. A., Gullion C. M., Brown J. B., Smith D. H. (2004). Longitudinal follow-up and outcomes among a population with chronic kidney disease in a large managed care organization. *Archives of Internal Medicine*.

[3] Fox C. S., Larson M. G., Leip E. P., Culleton B., Wilson P. W. F., Levy D. (2004). Predictors of new-onset kidney disease in a community-based population. *Journal of the American Medical Association*.

[4] Ueda H., Ishimura E., Shoji T. (2003). Factors affecting progression of renal failure in patients with type 2 diabetes. *Diabetes Care*.

[5] Bleyer A. J., Shemanski L. R., Burke G. L., Hansen K. J., Appel R. G. (2000). Tobacco, hypertension, and vascular disease: risk factors for renal functional decline in an older population. *Kidney International*.

[6] Muntner P., Coresh J., Smith J. C., Eckfeldt J., Klag M. J. (2000). Plasma lipids and risk of developing renal dysfunction: the atherosclerosis risk in communities study. *Kidney International*.

[7] Halimi J.-M., Giraudeau B., Vol S. (2000). Effects of current smoking and smoking discontinuation on renal function and proteinuria in the general population. *Kidney International*.

[8] Fried L., Solomon C., Shlipak M. (2004). Inflammatory and prothrombotic markers and the progression of renal disease in elderly individuals. *Journal of the American Society of Nephrology*.

[9] Shlipak M. G., Katz R., Kestenbaum B., Fried L. F., Siscovick D., Sarnak M. J. (2009). Clinical and subclinical cardiovascular disease and kidney function decline in the elderly. *Atherosclerosis*.

[10] Chonchol M., Gnahn H., Sander D. (2008). Impact of subclinical carotid atherosclerosis on incident chronic kidney disease in the elderly. *Nephrology Dialysis Transplantation*.

[11] Shimizu M., Furusyo N., Mitsumoto F. (2015). Subclinical carotid atherosclerosis and triglycerides predict the incidence of chronic kidney disease in the Japanese general population: results from the Kyushu and Okinawa Population Study (KOPS). *Atherosclerosis*.

[12] Takenouchi A., Tsuboi A., Terazawa-Watanabe M., Kurata M., Fukuo K., Kazumi T. (2015). Direct association of visit-to-visit HbA1c variation with annual decline in estimated glomerular filtration rate in patients with type 2 diabetes. *Journal of Diabetes and Metabolic Disorders*.

[13] American Diabetes Association (2015). (9) Microvascular complications and foot care. *Diabetes Care*.

[14] Matsuo S., Imai E., Horio M. (2009). Revised equations for estimated GFR from serum creatinine in Japan. *American Journal of Kidney Diseases*.

[15] Levey A. S., De Jong P. E., Coresh J. (2011). The definition, classification, and prognosis of chronic kidney disease: a KDIGO Controversies Conference report. *Kidney International*.

[16] Yanase T., Nasu S., Mukuta Y. (2006). Evaluation of a new carotid intima-media thickness measurement by B-mode ultrasonography using an innovative measurement software, intimascope. *American Journal of Hypertension*.

[17] Ito H., Komatsu Y., Mifune M. (2010). The estimated GFR, but not the stage of diabetic nephropathy graded by the urinary albumin excretion, is associated with the carotid intima-media thickness in patients with type 2 diabetes mellitus: A Cross-Sectional Study. *Cardiovascular Diabetology*.

[18] Lu B., Wan J., Yang Y., Li Y., Hu R. (2013). The estimated glomerular filtration rate is associated with subclinical atherosclerosis, independently of albuminuria, in patients with type 2 diabetes. *International Angiology*.

[19] Ishikaza N., Ishizaka Y., Toda E.-I. (2007). Association between chronic kidney disease and carotid intima-media thickening in individuals with hypertension and impaired glucose metabolism. *Hypertension Research*.

[20] Zhang L., Zhao F., Yang Y. (2007). Association between carotid artery intima-media thickness and early-stage CKD in a Chinese population. *American Journal of Kidney Diseases*.

[21] Preston E., Ellis M. R., Kulinskaya E., Davies A. H., Brown E. A. (2005). Association between carotid artery intima-media thickness and cardiovascular risk factors in CKD. *American Journal of Kidney Diseases*.

[22] Kawamoto R., Ohtsuka N., Kusunoki T., Yorimitsu N. (2008). An association between the estimated glomerular filtration rate and carotid atherosclerosis. *Internal Medicine*.

[23] Pang Y., Sang Y., Ballew S. H. (2016). Carotid intima-media thickness and incident ESRD: The Atherosclerosis Risk in Communities (ARIC) study. *Clinical Journal of the American Society of Nephrology*.

[24] Gorst C., Kwok C. S., Aslam S. (2015). Long-term glycemic variability and risk of adverse outcomes: a systematic review and meta-analysis. *Diabetes Care*.

[25] Ceriello A., Kilpatrick E. S. (2013). Glycemic variability: both sides of the story. *Diabetes Care*.

[26] Reddy M. A., Zhang E., Natarajan R. (2015). Epigenetic mechanisms in diabetic complications and metabolic memory. *Diabetologia*.

[27] Estacio R. O., Jeffers B. W., Gifford N., Schrier R. W. (2000). Effect of blood pressure control on diabetic microvascular complications in patients with hypertension and type 2 diabetes. *Diabetes Care*.

[28] Sone H., Tanaka S., Iimuro S. (2010). Long-term lifestyle intervention lowers the incidence of stroke in Japanese patients with type 2 diabetes: a nationwide multicentre randomised controlled trial (the Japan Diabetes Complications Study). *Diabetologia*.

